# Psychological Analysis of Religious Experience: The Construction of the Intensity of Religious Experience Scale (IRES)

**DOI:** 10.1007/s10943-020-01084-7

**Published:** 2020-09-09

**Authors:** Stanisław Głaz

**Affiliations:** Department of Philosophy, Institute of Psychology in Kraków, Jesuit University Ignatianum, Krakow ul. Kopernika 26, 31-501 Kraków, Poland

**Keywords:** Religious experience, Experience of God’s presence, Experience of God’s absence, Intensity of Religious Experience Scale (IRES)

## Abstract

The aim of this article is to present the issues of religious experience, and the associated experience of God’s presence and God’s absence, and then its operationalization, as well as to construct the Intensity of Religious Experience Scale, IRES (Skala Intensywności Doświadczenia Religijnego, SIDR). The value of psychometric tool, the reliability and validity, were assessed. The study was conducted in three steps. Study 1 concerned the generalization of statements related to conception of Catholic religious experience; i.e., the subjective feeling whether one experiences God’s presence and God’s absence, and how such beliefs affect certain aspects of a person’s life. Study 2 was carried out on the sample of 217 people and was designed to perform Exploratory Factor Analysis and to assess three-week test–retest reliability of the IRES. Study 3 was based on the sample of 368 people and was aimed to perform Confirmatory Factor Analysis and concurrent validation of the IRES. The analysis of the religious experience showed that this kind of human experience has its own structure. The explication of the subject has confirmed the existence of two positive factors of religious experience; i.e., a subjective sense of experience of God’s presence and God’s absence that can influence on life of people living in the Catholic religion environment.

## Introduction

With regard to religion, the term “experience” means the process of directly obtaining information about religious reality and the entirety of religious experiences of man (Głaz 2003). It is connected with various Christian religions and in particular in Catholicism (Głaz 1998). James (1902) and Starbuck (1899) analyzing religious experience, they noticed that it is characterized by inexpressibility, impermanence and at the same time emphasizing its emotional element but it depends on the person, and other circumstances. For them the religious experience is the unique experience of “something more”, “divinity”. According to Otto (1968), religious experience is associated with a sense of sanctity and dependence. Religious experience has a responsive and dialogical character, and not only cognitive. It is a personal encounter with God, Absolut (Argyle 2000). Religious experience—according to Jung (1982, 2010)—cannot be identified with what is psychological. Psychological factors could be only carriers of religious content.

The analysis of religious experience, shows that there are different kinds of it (Maslow 1970; Allport 1972). It may occur in two varieties that is as ordinary, and as mystical. The mystical experience is already knowing God, while the ordinary experience is also an encounter with God directly in prayer, even though one does not hear His answer (Głaz 1998). Płużek (1986) mentions several varieties of religious experience. A frequent type is searching for and finding paths to God. Another variation of religious experience is searching for God, feeling His presence, also related to the return of man to God. A specific religious experience is the experience of the presence of God and the absence of God (Głaz 1998).

Many researchers, when analyzing the effects of religious experience, point to its creative character (Keating 2001; Jung 2010), not including those who perceive the experience in reductionist term, as suggested by Freud (1961) and others (Master and Houston 1966). A man who participated in the religious experience perceives the world as friendlier to him (Oman and Thoresen 2005), feels more integrated, more uniform, more efficiently organized (Rogers 2012), more creative, and characterized by the uniqueness of the self (Rogers 1975). Religious experience helps in searching for the meaning of life. It releases man from the old paths of life (Azari et al. 2005), which favors a positive influence on the further development of personality and religiousness (Rydz et al. 2017). A man who has had the experience of the Absolut, is more diligent, has greater satisfaction with a job (Fromm 1966). Researchers show that the religious experience also have therapeutic implications. It is able to change the views of a person to themselves, their own lives, the world and to other people. It liberate more creative power in man (Płużek 1986). Religious people discover in themselves the feelings of gratitude to God. It is expressed in prayer, adoration, thanksgiving, sacrifice, love for others and even in commitments (Shear 2005).

In the 1960s, in order to better understand and describe the structure of the religious experience of man, researchers used the method called open-ended responses, which is qualitative measure. For example, Klingberg (1959) conducted a study among adolescents, who were asked to answer the question “Once when I thought about God…?” Others proposed to answer such questions like “Have you ever as an adult had the feeling that you were somehow in the presence of God?” (Glock and Stark 1965). When the qualitative method used in the description of religious experience demonstrated to be insufficient and incomplete, quantitative research tools have been developed. Among the claims about religious content, the researchers also included statements regarding religious experience. These instruments were based on a review of scientific literature, both from a psychological and theological perspective. For example, Underwood and Teresi (2002) constructed a tool for measuring ordinary and everyday spiritual experiences “Daily Spiritual Experiences Scale”. Some statements include content about the experience of God’s presence: „I feel God’s presence”, “I desire to be closer to God or in union with Him”. Hall and Edwards (2002) developed a multi-dimensional tool to assess the level of religious maturity of the individual “The Spiritual Assessment Inventory”. It also contains statements regarding religious experience like “I am aware of God’s presence in times of need”, “My experience of God’s presence impacts me greatly”.

## The Basics of the Intensity of Religious Experience Scale (IRES)

The basis of the scale of religious experience are the Catholic anthropology and the concept of God (Głaz 1998). The Catholic anthropology assumes that man is a person who fulfills his talents, predispositions, abilities in relationships with himself, with others and with God. Man, as an individual has a complex structure. It consists of the biological-physiological, psychological and spiritual dimensions (Frankl 1987). They work together. Each of them has an appropriate function. Bio-Physiological dimension includes the structural and functional integrity of the body. It includes impulsive dynamics and conditioning. The mental dimension is created by mental states such as experience, emotions and feelings. That is why man is open to the world, makes choices, and is creative. The spiritual dimension, which is the exclusive property of man, is an autonomous, dynamic factor. Thanks to its people get to know, reflect, analyze, evaluate, and make decisions. This dimension also directs man to God (Głaz 2013b). Man has the ontical ability to establish a personal relationship with God. The Catholic concept of God presents Him as a personal God who revealed Himself in Christ, sending out the Holy Spirit, and entering into relationships with men (Jarosz 2011).

Literature shows that many pious people have experienced the presence of God and the absence of God (James 1902; Saint Jean de la Croix 1915). The both religious experiences can be noted in the lives of our contemporaries (Głaz 2011, 2014). They are connected with the process of development of religious life (Głaz 1998). During the experience of God’s presence, man is aware of His presence in his life or His omnipresence in the world. This kind of experience is connected a feeling of joy, an elation, a feeling of surprise, and a feeling of inner calmness. It helps to deepen the relation with God and with other people. It also deepens the faith and love to God. Man who is convinced of God’s presence, can however experience His temporary absence. Sometimes it is accompanied by the inner emptiness and loneliness. It evokes feelings of dissatisfaction, doubt and sometimes even rejection by God (Głaz 1998, 2014). During the experience of God’s absence, man experiences such features as lack of self-confidence, lack of patience, lack of understanding for oneself and others. In fact, it can be the greatest trial of faith because in spite of these appearing negative features, there are also such features as a desire to deepen faith, greater trust in God, greater involvement in religious life, awareness of own imperfections, appreciation of God’s presence (Głaz 1998, 2013b). Such internal and direct experiences can be perceived only by the experiencing man, and from the outside one can be people who experience it, and it can be observed from the outside by human behaviors. Awareness of these religious experiences may accompany a person for a longer or shorter time, in various stages of their life’s development (Eigen 2001; Głaz 2014). Every kind of religious experience, that is the presence of God and the absence of God, brings something good in the life of a given person, and it given him or her a new content. It leaves a certain trace in the person’s life. Its effects are visible in life in the personal and social sphere, as well as in religious and psychological behaviors. The religious experience of the presence of God and the absence of God is integrated into the totality of human experience (Jung 2010). The cognitive, emotional and value dimension of a human being participates in it. It has a personal character, although it can be socially and culturally conditioned taking into account religious differences (Głaz 1998; Huber 2003). On the basis of such an understanding of religious experience, an attempt was made to construct a scale of the religious experience: God’s presence as well as God’s absence.

### Construction of the IRES

Many factors indicate the need to construct the scale relating to the concept of Catholic religious experience: the experience of God’s presence and the experience of God’s absence.

We want to know to what extent the proposed concept of Catholic religious experience of subjective feeling of God’s presence and God’s absence influencing some life aspects is scientific, true and verifiable.

The motivation to construct a tool for measuring the experience God’s presence and God’s absence is the increase in religious awareness and the usefulness of religion in the life of Poles. The Institute of Statistics of the Catholic Church in Poland (2015) stated that over 90% of Polish citizens declare membership of the Roman Catholic Church. After political changes, there is a great interest among believers and doubters about religious movements and communities, as well as religious press. Every tenth Pole considers his faith as deep. Many people declare that they have experienced the presence and absence of God in their lives (Głaz 2011, 2014). There is an increase in the participation of believers in the Sunday liturgy and the Eucharist in the last decade. There is also a visible departure from institutional religiosity in favor of intrinsic religiosity (Mariański 2011, 2014; Głaz 2013b; Szyszka 2018).

Researchers developed standardized tools for studying religiosity, taking into account the dimension of Christian religious experience (Hill and Hood 1999). Foreign language adaptations of this type of tools to Polish conditions were also made (Zarzycka 2007; Zarzycka and Bartczuk 2011). However, there is still a lack of a standardized and satisfactory tool for measuring Catholic religious experience. It seems that the present tool for studying Catholic religious experience: God’s presence and God’s absence will to some extent fill the gap in this area.

The Scale of Religious Experiences (SRE) contains 37 items (Głaz 2011). Only Exploratory Factor Analysis was performed, but no Confirmatory Factor Analysis was carried out (validity of the scale). It has three subscales: Religious experience, Experience of God’s presence, and Experience of God’s absence (Głaz 2011). During the examination, participants often pointed out that there is too many items, which sometimes caused troubles. It was decided to construct a scale with two factors experience of God’s presence as well as God’s absence. While constructing the scale, it was decided to abandon the Religious experience subscale.

The existing tools seem insufficient and require further expansion. This mainly concerns taking into account the specificity of Catholic religious experience: God’s presence and God’s absence, as well as indicating the positive role of these two aspects in the life of modern man.

In the development of tools for the study of religious experience, the merits of the researchers are undisputed. However existing tools have limited applications. The development of a tool to study Catholic religious experience: God’s presence and God’s absence has a comprehensive approach. It seems that thank to the tool a new aspects of the Catholic experience in relation to the various elements of human life can be investigated.

Thus, the main purpose of this article is, on the basis of the concept of religious experience, to show construct the tool containing two subscales measuring the experience of God’s presence and the experience of God’s absence of people living in the Catholic religion environment that have an influence on the cognitive, emotional, and behavioral spheres of human life in the religious and mental dimensions.

The aim of the research procedure is threefold: 1. Confirmation of the specificity of the Catholic religious experience of God’s presence and God’s absence in relation to human life; 2. The construction of the Intensity of Religious Experience Scale (IRES); 3. Determining the psychometric value of the scale.

### Validation of IRES

The validation of the IRES was done in the three study steps. Study 1 was to collect statements related to the concept of Catholic religious experience of God’s presence and God’s absence, evaluating them, and generating items for further analysis. Study 2 was carried out to perform the Exploratory Factor Analysis and to assess the three-week test–retest reliability of the IRES. Study 3 was conducted out to perform the Confirmatory Factor Analysis.

### Study 1: Pilot Study

In order to test the generated primary IRES statements, i.e., to clarify them to the adapted concept of Catholic religious experience of God’s presence and God’s absence, and to verify whether the individual statements actually measure what is intended to be measured, and whether they are understandable and correctly understood, pilot study were conducted.

The Scale of Religious Experience (SRE) (Głaz 2011) was used as the starting point for the development of the IRES scale. The SRE contains 37 items. As mentioned earlier, this scale is related to the Catholic religious experience. In its construction, these items whose loadings were higher than .400 were considered. The grouped homogeneous items and the list of loadings in the columns of factor matrices allowed for the constitution of three factors. They concern: 1. Religious experience, 2. Experiencing God’s presence, and 3. Experiencing God’s absence. The Cronbach’s alpha coefficient of reliability for individual factors is .89 ≤ *α* ≤ .91. No confirmatory factor analysis was performed. It contains an unequal number of items in the factors. Therefore, it was decided to rephrase some items in the construction of the new version of the scale, and to give up some of the items and introduce others. It was also decided to exclude all the items constituting the first factor regarding religious experience, due to its inadequacy with the intended subscales.

The collected material related to the concept of Catholic religious experience of God’s presence and God’s absence obtained through surveys, in pastoral work, during interviews and taken from the scientific literature, as well as from the previous scale (SRE), served to develop an initial pool of items for the IRES. First of all, the statements were evaluated by a group of people in scientific seminars. Those statements, the content of which was similar and incomprehensible, were dropped out. Some of them were corrected. We generated a list of face-valid 31 statements that were accepted for analysis. Then the accuracy of each statement was evaluated by a group of theologians and psychologists and on its basis the final selection of the statements was made. According to the method proposed by Lawshe’s (1975), 31 prepared statements were evaluated on a three-point scale (3 = *this statement is essential for a given scale*, 2 = *this statement is useful, but it is not essential for a given scale*, 1 = *this statement should not be in a given scale*). The statements (10 statements) which, according to experts, demonstrated to be useless, ambiguous, were excluded. 21 statements were accepted for further analysis. Each of them has 7 possible answers. 7 = *definitely yes* (*I definitely agree*), 6 = *yes* (*I agree*), 5 = *rather yes* (*I rather agree*), 4 = *I cannot decide*, 3 = *rather not* (*I rather don’t agree*), 2 = *no* (*I do not agree*), 1 = *definitely not* (*I definitely disagree*). The generated items for the future tool were subject to a further test procedure aimed at verifying its psychometric properties.

### Study 2: Exploratory Factor Analysis and Three-Week Test–Retest Reliability of the IRES

After 21 statements related to the concept of Catholic religious experience of God’s presence and God’s absence was generated, the next step of work on the tool was to use exploratory factor analysis and three-week test–retest reliability of the IRES to determine an empirical verification of the scale validity. Based on the adapted conception of Catholic religious experience, a two-factorial research tool is expected, and subscales would have Cronbacha’s alpha coefficients sufficiently high.

### Method

#### Participants and Procedure

The group consisted of students at the Higher School of Insurance in Krakow, as well as Poles working in England in the town of Lincoln. People were asked about their place of birth, belonging to a religious group, as well as religious commitment. All respondents were born in Poland and grew up in a Catholic family. They consider themselves believers and practitioners. They take part in the Sunday Eucharist, they pray and take sacraments. They had in their lives the experiences of God’s presence and God’s absence. In order to obtain material for the further construction of the scale, people were asked to respond to each statement. The subject’s task was to express an opinion on a 7-point Likert-type scale, about the extent in which he or she agrees with or does not agree with the content of the statement. The analysis excluded those of people who did not respond to all test items. There were 10 of them. For further analysis, the results obtained on the basis of 217 correctly completed sets of questionnaires of people were used. The age of the respondents ranged from 23 to 47 (*M* = 39.2; *SD* = 9.18). 22.3% of the surveyed population were men and 77.7% women.

### Results

#### Exploratory Factor Analysis of the IRES

In order to check whether the previously collected set of items related to the concept of Catholic religious experience of God’s presence and God’s absence has diagnostic power, it was evaluated by the method of competent judges, and then developed. The obtained data carried out in the sample were subjected to exploratory factor analysis. The separated factors were subjected to oblique rotation. According to the theory and adapted hypothesis, it was decided to use a two-factor solution. To determine the factual validity, the results were analyzed using the rotation method—Varimax. Matrix of factor loadings is included in Table 1.

**Table 1:** Factors Loadings of the items of the IRES (*N* = 217)

Items	The content of items regarding the subscale: experience of God’s presence (PG)	*F*1
13	During the experience of God’s presence God reveals Himself to me as someone in whom it is worth trusting. *W trakcie przeżycia obecności Boga, Bóg objawia się mi jako ktoś, w kim warto pokładać ufność*	.868
11	The experience of God’s presence is conducive to deepening my religious faith. *Przeżycie obecności Boga sprzyja pogłębieniu mojej wiary religijnej*	.842
9	I think that my experience of God’s presence promotes a better understanding of myself. *Myślę, że moje przeżycie obecności Boga sprzyja lepszemu poznaniu samego siebie*	.838
7	The experience of God’s presence is conducive to deepening the intimacy with Him. *Przeżycie obecności Boga sprzyja pogłębieniu zażyłości z Nim*	.833
12	Experiencing God’s presence opens me more to the needs of other people. *Przeżycie obecności Boga bardziej otwiera mnie na potrzeby innych ludzi*	.826
16	Experiencing God’s presence leaves a sense of delight and admiration in me. *Przeżycie obecności Boga pozostawia we mnie poczucie podziwu i zachwytu*	.823
3	During the inner experience of God’s presence, I experience states of deep peace and joy. *Podczas wewnętrznego przeżycia obecności Boga doświadczam stanów głębokiego pokoju i radośc*i	.793
1	The experience of God’s presence is for me a source of strength in the daily hardships of life. *Przeżycie obecności Boga jest dla mnie źródłem siły w codziennych trudach życia*	.783
18	During the experience of God’s presence, God reveals Himself to me as the one who is difficult to describe in words. *W trakcie przeżycia obecności Boga, Bóg objawia się mi jako Ten, którego trudno opisać słowami*	.736
2	Thanks to the experience of the presence of God, the lives seems to be more meaningful and valuable. *Dzięki przeżyciu obecności Boga życie wydaje mi się bardziej sensowne i wartościowe*	.528

Items	The content of items regarding the subscale: experience of God’s absence (AG)	*F***2**
17	Experiencing God’s absence is conducive to deepening my trust in Him. *Przeżycie nieobecności Boga sprzyja pogłębieniu mojej ufności wobec Niego*	.820
14	During the experience of God’s absence, I have the opportunity to better understand myself. *Podczas przeżycia nieobecność Boga, mam możliwość lepszego zrozumienia samego siebie*	.796
15	Thanks to the experience of the absence of God, I am more open to personal encounters with others. *Dzięki przeżyciu nieobecności Boga, jestem bardziej otwarty na osobiste spotkania z drugimi*	.794
8	Thanks to the experience of God’s absence, I am able to understand more the involvement of others in relation to other’s the expectations. *Dzięki przeżyciu nieobecności Boga jestem w stanie bardziej zrozumieć zaangażowanie drugich osób względem oczekiwań innych*	.761
6	I think that the experiencing the God’s absence provides me with new knowledge about others. *Myślę, że przeżycie nieobecności Boga dostarcza mi nowej wiedzy o innych*	.745
5	Experiencing the God’s absence enriches my religious life. *Przeżycie nieobecności Boga ubogaca moje życie religijne*	.731
10	Although in my life the experience of God’s absence appears, I see God as the one who releases creative anxiety in me. *Pomimo że w moim życiu pojawia się przeżycie nieobecności Boga, to Boga postrzegam jako tego, który wyzwala we mnie twórczy niepokój*	.659
4	Although in my life there is an experience of the God’s absence I see God as the one who gives meaning to every life. *Mimo to że w moim życiu pojawia się przeżycie nieobecności Boga, to Boga postrzegam jako tego, który nadaje sens każdemu życiu*	.461

The variables (statements) whose loadings were higher than .450 were taken into account. 18 statements met these criteria (Table 1). Three statements were excluded because they had factor loadings lower than .450. One statement concerned the experience of God’s presence “During the experience of God’s presence, God reveals his intentions to man” (.411), and two statements concerned the experience of God’s absence. “I think the experience of God’s absence leaves in me the feeling of anxiety and uncertainty” (.401) and “Although in my life I experience the absence of God, I see God as the one who cares for me” (.364). The grouped statements of similar content, homogeneous and the combination of loadings in the columns of factor matrices, formed the basis for determining the name for particular factors, namely the first factor concerns the experience of God’s presence (PG), in which there were 10 statements, and the second factor concerns the experience of God’s absence (AG), which consists of 8 statements (see Table 1). Factor I, explains 33.1% of the variance of the experience of God’s presence, and the factor II accounts for 22.3% of the variance of the experience of God’s absence. The factors loadings of the items are strong. The obtained factors are correlated with each other. The obtained correlation coefficient between the separated factors is: *r* = .39, *p* < .01. This suggests that the accepted statements of the scale related to Catholic religious experience of God’s presence and God’s absence, and the isolated factors confirm the structure of religious experience and form the basis for further analyzes.

#### Reliability Analysis of the IRES

The reliability of the tool informs the researcher how far the statements included in the scale are similar to each other, whether they measure the same construct, in this case the experience of God’s presence and God’s absence. Verification of the reliability of the subscales were estimated using the internal coherence and absolute stability methods. In order to determine the internal coherence of the scale, Cronbach’s α coefficient and Guttman’s λ_6_ for two subscales were calculated. Table 2 presents descriptive statistics and reliability indicators for both subscales.

**Table 2:** Descriptive statistics and reliability indicators for two subscales of the IRES (*N* = 217)

Subscales	*M*	*SD*	*α*	*λ*_6_
Experience of God’s presence (PG)	5.2	1.21	.93	.91
Experience of God’s absence (AG)	4.1	1.09	.86	.85

The reliability indexes of the experience of God’s presence (PG) and the experience of God’s absence (AG) are high (Table 2). Cronbach’s values for both subscales are satisfactory, which shows a high reliability of the measure as well as Guttman’s do. The factor PG reveals a greater saturation of the contents related to the experience of God’s presence than the factor AG concerning the experience of God’s absence.

The stability of the subscales was estimated by a test–retest method with an interval of three weeks. The results are shown in Table 3. The average results obtained from the test and retest are very similar. The obtained measures of correlation between the results from the first and second studies confirmed the stability of the scale. Both factors have a high stability indicator. For the subscale of the experience of the presence of God (PG), the correlation coefficient is *r *= .83, *p* < .001, and for the subscale of the experience of absence of God (AG), it is *r* = .80, *p* < .001.

**Table 3:** Descriptive statistics and correlations between the first and second research for the IRES (*N* = 60)

Subscales	I study		II study		*r*
	*M*	*SD*	*M*	*SD*	
Experience of God’s presence (PG)	5.4	.989	5.1	1.09	.83***
Experience of God’s absence (AG)	3.9	1.11	4.2	1.01	.80***

***p < .001

### Study 3: Confirmatory Factor Analysis and Concurrent Validation of the IRES

After exploratory factor analysis and reliability of the IRES, to check the adequacy of the hypothetical model, whether the factor structure of the scale from Study 2 would replicate in another sample, validity of the scale was done through the use of confirmatory factor analysis and criterion validity of the scale by applying the correlation method. It was assumed that a confirmatory factor analysis model with two-correlated factors would show adequate fit and that subscales would correlate moderately with factors of similar content.

### Method

#### Participants and Procedure

The analysis was carried out on the results obtained among the group of students consisted of full-time and part-time of Jesuit University Ignatianum in Krakow. People were asked about their place of birth, religious commitment, as well as belonging to a religious group. All respondents were born in Poland and grew up in a Catholic family. They consider themselves practitioners and believers. They take part in the Sunday Eucharist, take sacraments and they pray. They declared that they had in their lives the experience of God’s presence and God’s absence. The analysis excluded those of people who did not respond to all test items. There were 27 of them. For further analysis, the results obtained on the basis of 368 correctly completed sets of questionnaires of people were used. The age of the participants ranged between 19 and 23 (*M* = 20.16; *SD* = 3.19). 14.1% of the surveyed population were men and 85.9% women.

### Measures

- Participants completed the final IRES version, 18-item scale as developed and described in previous Study 2. The persons under study expressed their opinions on a 7-point Likert-type scale, how they agreed or disagreed with the content of the statements. The Cronbach’s alpha coefficients for the current study were .93 for God’s presence subscale and .87 for God’s absence subscale. The factor associated with the experience of God’s presence reveals greater satiation of the contents related to the experience of God’s presence (*M* = 5.3; *SD* = 1.08) than the factor concerning the experience of God’s absence (*M* = 4.0; *SD* = 1.12). In addition, participants completed the following measures:
- The Personality Inventory (NEO-FFI) by McCrae and Costa (1997) used to determine the relatively constant personality traits. In McCrae and Costa’s view (1997) the distinguished traits really exist. They are significant for the individual’s adaptation to the environment. They are universal and stable, meaning independent of one’s race, cultural background, or gender, and biologically determined. The answer to the 60 items of the NEO-FFI questionnaire allows to obtain information on five basic personality dimensions such as: Neuroticism, Extroversion, Openness, Agreeableness, Conscientiousness. The subject’s task is to express an opinion according to a 5-point Likert scale. 5 = *definitely yes*, 4 = *rather yes*, 3 = *I cannot decide*, 2 = *rather not*, 1 = *definitely not*. Adaptations to Polish conditions were made by Zawadzki et al. (1998). The scale has satisfactory reliability (.68 ≤ *α* ≤ .82) and theoretical validity.
- The Purpose in Life Test (PIL) by Crumbaugh and Maholick (1964) was used to assess the meaning of life. Crumbaugh and Maholick used Frankl’s existential ideas from logotherapy to assist in the development of their test. Frankl defined meaning in life as the ontological significance of life from the point of view of the experiencing individual (Frankl 1987). The test contains 20 statements. The subject’s task is to express an opinion according to a 7-point Likert-type scale, concerning the extent that one agrees with or does not agree with the content of the claim. 7 = *definitely yes*, 6 = *yes*, 5 = *rather yes*, 4 = I *cannot decide*, 3 = *rather not*, 2 = *no*, 1 = *definitely not*. Popielski (1987) made an adaptation of the scale to Polish conditions. The scale has satisfactory reliability (α = .90) and theoretical validity. The high overall score of the scale indicates a high level of sense of life, a low score—a low level of sense of life.
- The Scale of Personal Religiosity (SPR) by Jaworski (1989) used to measure Catholic religiosity. It contains 30 statements. The subject’s task is to express an opinion according to a 5-point Likert-type scale, concerning the extent that one agrees with or does not agree with the content of the item. 5 = *definitely yes*, 4 = *rather yes*, 3 = *I cannot decide*, 2 = *rather not*, 1 = *definitely not*. The scale has four factors: I—religious faith (WR), II—morality (MR), III—religious practices (PR), IV—religious self (SR). The correlation coefficient between the factors is positive and ranges from .51 to .62. Cronbach’s alpha coefficient is: .78 ≤ *α* ≤ .89.
- The Centrality of Religiosity Scale (CRS) by Huber was used to measure Christian religiosity (Huber and Huber 2012). The scale contains 15 questions. The subject’s task is to answer each question by choosing: 1 = *not at all*, 2 = *slightly*, 3 = *on average*, 4 = *rather*, 5 = *very*. The scale defines five dimensions of Christian religiosity: interest in religious issues—the intellectual dimension (ZPR), religious beliefs—dimension of ideology (PR), prayer—dimension of private practice (M), religious experience—the dimension of religious experience (DR) and worship—dimension of public practice (K). Adaptation to Polish conditions was made by Zarzycka (2007). The reliability of the scale is estimated using Cronbach’s alpha and is: .82 ≤ *α* ≤ .90. The obtained measures of correlation between the results obtained in the first and second studies confirmed the stability of the scale (*r* = .62–*r* = .85).
- The Scale of Personal Relationship to God (SPRdB) by Jarosz (2011) was used to measure the relationship to God. It contains 20 statements. The subject’s task is to express an opinion according to a 5-point Likert-type scale, concerning the extent that one agrees with or does not agree with the content of the item. 5 = *definitely yes*, 4 = *rather yes*, 3 = *I cannot decide*, 2 = *rather not*, 1 = *definitely not*. This scale defines three types of relationships to God. Updated relationship (RZ), mutual relationship (RW), dialogue relationship (RD). The obtained measures of correlation between the results from the first and second tests confirmed the stability of the scale (*r* = .66–*r* = .84). The reliability coefficient (Cronbach’s alpha) for individual factors is: .79 ≤ *α* ≤ .92.

## Results

### Validity of the IRES

**Fig. 1:**
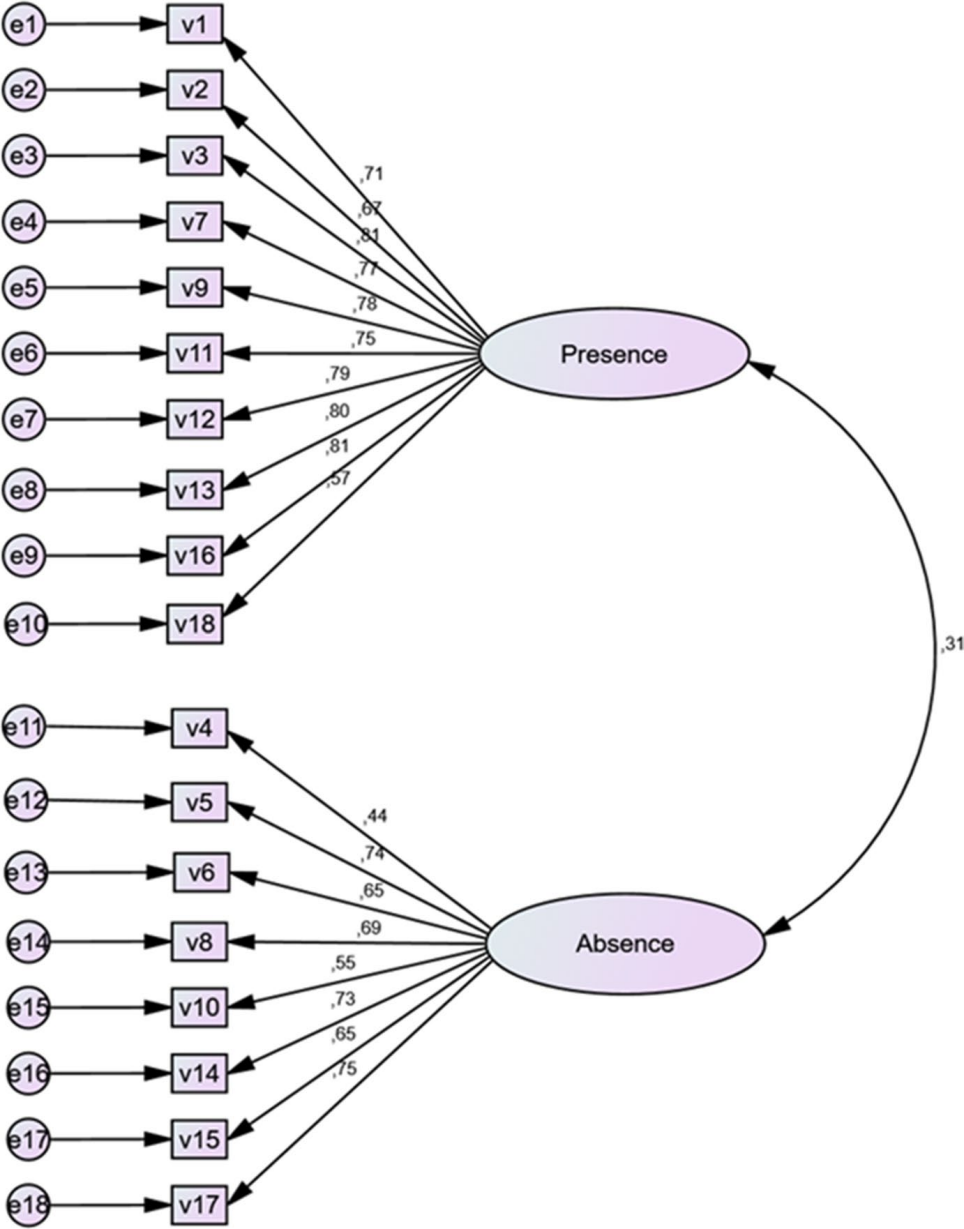
The factor confirmation analysis model for the IRES. Above the arrows, the size of the factor loadings of the scale items has been placed (*N *= 368)

The hypothesis was assumed that the confirmatory factor analysis model with two-correlated factors would show adequate fit. The values of the scale parameters were estimated and verified by the Maximum Likelihood (MLR) method by means of confirmatory factor analysis performed using the SPSS Amos package. The properties of the confirmatory factor analysis model are presented in Table 4, and the size of the loadings of individual statements is shown in Fig. 1.

**Table 4:** Coefficients of goodness of fit in the model of confirmatory factor analysis of the IRES (*N* = 368)

Two-factor model	CMIN/df	CMIN/X^2^	GFI	AGFI	CFI	RMSEA
	2.06	3.62	.93	.91	.95	.06

*CFI* comparative fit index, *AGFI* adjusted goodness of fit index, *GFI* goodness of fit index, *RMSEA* root mean square error of approximation

Absolute indices of goodness of fit were used to evaluate the model. Criteria indicated in Table 4 were assumed. Examining all the indices of goodness of fit considered in this study the values of the adapted model fit indicators for the tested model have proved satisfactory including RMSEA = .06 (Marsh and Hocevar 1985). Based on the obtained results, it can be assumed that the factor validity of the subscales has been confirmed. The obtained correlation coefficient between the separated factors is: *r* = .31, *p* < .01. This suggests that the structure of the scale is well-defined and plausible. It also indicates that the scale meets the required psychometric parameters.

### Criterion Validity of the IRES

The criterion validity of the scale was checked and established by the method of correlation of values obtained in the scale by means of other research tools. It was expected according to the literature of the subject, as well as earlier studies (Głaz 2013a, 2014; Krok 2015), that the experience of God’s presence and the experience of God’s absence have a significant statistical and positive relationship with such personality traits as conscientiousness, openness, agreeableness, the meaning of life, and religiosity. Hence it was decided to show the strength and type of relationship between results obtained in the IRES and the Personality Inventory (NEO-FFI), the Purpose in Life Test (PIL), the Scale of Personal Religiosity (SPR), the Centrality of Religiosity Scale (CRS) and the Scale of Personal Relationship to God (SPRdB). In order to determine the strength of the relationship and its character between the variables taken in this work, Pearson’s correlation coefficient *r* was calculated. The results obtained in the scales shows Tables 5 and 6.

**Table 5:** Pearson’s *r* correlation values obtained for the IRES, the NEO-FFI) and the PIL (*N* = 368)

Variables	*N*	*E*	*O*	*A*	*C*	PLT
PG	− .02	.12*	.28**	.20**	.12*	.32**
AG	− .06	.04	.13*	.01	.13*	.18**

*PG* experience of God’s presence, *AG* experience of God’s absence, *N* neuroticism, *E* extraversion, *O* openness, *A* agreeableness, *C* conscientiousness, *PIL* the meaning of life

*p < .05; **p < .01

**Table 6:** Pearson’s *r* correlation values received for the IRES, the SPR, the CRS and the SPRdB (*N* = 368)

Variables	WR	MR	PR	SR	ZPR	PR	M	DR	K	RZ	RW	RD
PG	.73***	.72***	.74***	.71***	57**	.61**	.55**	.64**	.59**	.37**	.20**	.16*
AG	.22**	.23**	.23**	.22**	.31**	.22**	.17**	.28**	.21**	.15*	.12*	.11*

*PG* experience of God’s presence, *AG* experience of God’s absence, *WR* religious faith, *MR* morality, *PR* religious practices, *SR* religious self, *ZPR* interest in religious issues, *PR* religious beliefs, *M* prayer, *DR* religious experience, *K* worship, *RZ* updated relationship, *RW* mutual relationship, *RD* dialogical relationship

*p < .05; **p < .01; ***p < .001

The analysis of the results obtained in the IRES, in the NEO-FFI and in the PLT shows (Table 5) that a statistically significant relationship and positive occur between the experience of God’s presence (PG) and extraversion (E) (*r* = .12, *p* < .05), openness (O) (*r* = .28, *p* < .01), agreeableness (A) (*r* = .20, *p* < .01), conscientiousness (C) (*r* = .12, *p* < .05), and also between the experience of God’s absence (AG) and openness (O) (*r* = .13, *p* < .05) and conscientiousness (C) (*r* = .13, *p* < .05). Moreover, a significant statistical relationship is recorded between the experience of God’s presence (PG) (*r* = .32, *p* < .01) and the experience of God’s absence (AG) (*r* = .18, *p* < .01) and the sense of meaning in life (PIL).

Explanation of the results obtained in the scales: IRES, SPR, CRS, and SPRdB shows (Table 6) that the statistically significant relationship and positive is observed between the experience of God’s presence (PG) and religious faith (WR) (*r* = .73, *p* < .001), morality (MR) (*r* = .72, *p* < .001), religious practices (PR) (*r* = .74, *p* < .001), religious self (SR) (*r* = .71, *p* < .001), and self-forgiveness (PS) (*r *= .31, *p* < .01). In addition, between the experience of God’s absence (AG) and religious faith (WR) (*r* = .22, *p* < .01), morality (MR) (*r* = .23, *p* < .01), religious practices (PR) (*r* = . 23, *p* < .01), religious self (SR) (*r* = .22, *p* < .01), and self-forgiveness (PS) (*r* = .13, *p* < .05). Also, a statistically significant and positive relationship occurs between the experience of God’s presence (PG) and interest in religious issues (ZPR), (*r* = .57, *p* < .01), religious beliefs (PR) (*r* = .61, *p *< .01), prayer (M) (*r* = .55, *p* < .01), religious experience (DR) (*r* = .64, *p* < .01), worship (K) (*r* = .59, *p* < .01), as well as an updated relationship (RZ) (*r* = .37, *p* < .01), mutual relationship (RW) (*r* = .20, *p* < .01) and dialogical relationship (RD)) (r = .16, p < .05). In addition, between the experience of God’s absence (AG) and interest in religious issues (ZPR) (*r* = .31, *p* < .01), religious beliefs (PR) (*r* = .22, *p* < .01), prayer (M) (*r* = .17, *p* < .01), religious experience (DR) (*r* = .28, *p* < .01), worship (K) (*r* = .21, *p* < .01), as well as an updated relationship (RZ) (*r* = .15, *p* < .05), mutual relationship (RW) (*r* = .12, *p* < .05) and dialogical relationship (RD) (*r* = .11, *p* < .05).

## Discussion

The aim objective of the article was to present the construction a simple and psychometrically sound scale to study the intensity of religious experience: experience of God’s presence and God’s absence people living in the Catholic religion environment, that is a subjective feeling which is not examined in itself, but as the impact of that feeling on life. The article also presents the basis of the tool and rationale of its construction. The Intensity of Religious Experience Scale (IRES) has 18 items. There are two subscales. The first one consists of ten items (1, 2, 3, 7, 9, 11, 12, 13, 16, 18) and describes the intensity of the experience of the impact of the subjective conviction of feeling God’s presence on certain aspects of life (PG). The second subscale consists of eight statements (4, 5, 6, 8, 10, 14, 15, 17) and describes the impact of the subjective conviction of feeling God’s absence on certain aspects of life (AG).

Verification of the validity of the IRES scale and the extent to which the scale measures the adapted Catholic concept: the experience of God’s presence and God’s absence, was evaluated in three ways. The first step consisted in developing and generating statements related to the Catholic concept of religious experience of God’s presence and absence taken on. The second step concerned the use of the exploratory factor analysis, which was done and presented earlier (see results Table 1). Third method concerns the estimation of the validity of the scale through the use of confirmatory factor analysis (see results Fig. 1 and Table 4) and criterion validity of the scale by applying the correlation method (see results Tables 5 and 6). The validation process confirmed the IRES 18-item instrument, as valuable sound psychometric tool.

The IRES confirms that many of contemporary persons experiences God’s presence and God’s absence. According to the theory of Catholic religion (Głaz 1998) and the analysis of the structure of Catholic religious experience, it should be stated that within this experience there are two factors of it: God’s presence and God’s absence as subjective feelings.

The exploratory factor analysis method shows that the experience of God’s presence has a greater saturation with content related to this kind of experience than the experience of God’s absence. In the subscale of the experience of God’s presence, items have stronger factor loadings than items in the subscale of the experience of God’s absence. The mean value for the subscale of experience of God’s presence is higher than the average experience of God’s absence, as well as reliability measures, Cronbach’s and Guttman’s values. This indicates that in the Catholic religious experience being analyzed the experience of the presence of God is more important than the experience of God’s absence, and it plays a greater positive role in the life of the individual, which was also confirmed by previous studies (Głaz 2013a, b).

The validity of the scale was done through the use of confirmatory factor analysis and criterion validity of the scale by applying the correlation method.

The confirmatory factor analysis shows that the factors loadings of the items are strong. Examining all the indices of goodness of fit considered in this study, including RMSEA, it should be stated that the proposed two-factor model of religious experience is satisfactory. In addition, the magnitude of loadings of confirmatory factor analysis for particular statements (.44–.81; Fig. 1) is similar to loadings obtained by the exploratory factor analysis (.461–.868; Table 1), what shows that the scale has satisfactory structure.

Criterion validity of the IRES was determined by the correlations with other adequate tools as The Personality Inventory (NEO-FFI), the Purpose in Life Test (PIL), The Scale of Personal Religiosity (SPR), The Centrality of Religiosity Scale (SCR) and The Scale of Personal Relationship to God (SPRdB).

Analysis of correlation indicated that there is a positive correlation between the experience of God’s presence and the experience of God’s absence, and certain personality traits measured with the NEO-FFI and the PIL. As expected according to previous research (Głaz 2013b), it was confirmed that the sense of the influence of God’s presence or absence on one’s life has a statistically significant and positive relationship with conscientiousness and openness, moreover, the experience of God’s presence is related to extraversion and agreeableness. The correlation coefficient is: *r *= .12–*r *= .28. There is no such relationship between the experience of God’s presence and His absence with neuroticism. Both dimensions of religious experience have a significant positive relationship with the meaning of life (*r *= .18–*r *= .32). Directions of the obtained correlations are in line with expectations, positive, but the obtained values of correlation coefficients are low.

The IRES correlates statistically significantly and positively with all dimensions of the SPR. The correlation coefficients of these dimensions of religiosity for the experience of God’s presence range from .71 to .74, while for the experience of God’s absence from .22 to .23. Similar values as to the correlation coefficients are recorded between the IRES and the SCR. The magnitude of correlation coefficients for the experience of God’s presence ranges from .55 to .64, and for the experience of God’s absence from .17 to .31.

The smallest correlation coefficients are observed between the IRES and the SPRdB. Correlation coefficients for the experience of God’s presence range from .16 to .37, and slightly lower for the experience of God’s absence. The small values obtained for correlation coefficients between the dimensions of the IRES and some parameters of religiosity of the SPRdB may result from the content difference of the measured dimensions of religiosity. The IRES measures a sense of influence of God’s presence or absence on one’s life. The SPRdB includes religious dimensions such as worship, religious commitment, faith, religious self, etc., with the exception of religious experience included in the SCR, and defines religious content slightly different from those included in the IRES. Stronger positive relationships were expected between the results of the IRES and the results obtained in the SPRdB. Nevertheless, it should be admitted that there are statistically significant and positive correlations between the experience of God’s presence and the experience of God’s absence, which is an important component of religiosity, and the presented parameters of human religiousness, which confirms the value of the scale.

The undertaken analysis rises some comments.

In the validation process of the tool exploratory and confirmatory factor analysis techniques were used, that produced an 18-item instrument, which exhibits sound psychometric properties. Meanwhile, each of the factors may still be strengthened through revision, by adding new items that are not included in the present study.

The samples in the present investigation are representative for the Catholic population of student age. Thus, cautions should be taken when generalizing the findings despite the strong evidence of reliability and validity of the instrument.

Many tools have been developed to study religiousness, including religious experience, and their positive impact on human life has been analyzed (Hill and Hood 1999; Hall and Edwards 2002; Huber and Huber 2012). Empirical verification of the Catholic concept of religious experience: God’s presence and God’s absence confirmed that contemporary man manifests tendencies to realize himself on a spiritual and psychological level.

The tool in question does not examine experience, but a sense of the influence of the presence of God and absence of God on one’s own life taking into account their positive aspect. According to Catholic theology, also feeling of the experience of God’s absence can play a positive role in the life of a believer, which has been confirmed (Jaworski 1989; Głaz 1998, 2013b).

There are many measures that show that Christian religiosity has a great influence on psychological well-being in the life of the believers (Jarosz 2011; Huber and Huber 2012; Krok 2015), none of these indices investigated the possible relationship between Catholic subjective belief about the feeling of influence of God’s presence and God’s absence on certain aspects of one’s life and psychological outcomes for example, the meaning of life, and satisfaction in life.

## Conclusion

The IRES can be used among young people in the student age and maybe to older people as well, living in the Catholic religion environment. It can be used to diagnose how persons perceive their religious sense of God’s presence and God’s absence as affecting their life. It seems that the scale may be the most useful in studying individuals and religious groups that lead an intense religious life. The statements contained in it relate to certain aspects of deepened and mature religious life of an individual with its consequences regarding his personal and social life.

The test may have various applications, e.g., it can be a form of self-esteem in groups of young people to help guide their religious development. In high school or student groups, such a religious orientation can be helpful to young people, and for pastoralists leading such groups. Pastoral psychology presupposes the interpenetration of spiritual and mental development. Due to its nature and purpose, it can serve as a direct aid in pastoral practice of youth.

The both IRES subscales have a positive meaning and can be interpreted as measures of the sense of God’s presence and God’s absence as encouraging some aspects of human life. Thus, the high scores in the subscales of the experience of God’s presence and God’s absence can be interpreted positively as follows.

- The high score in the subscale of the experience of God’s presence (PG) suggests that such person has confidence in God, and seeks to have a relationship with Him. He or she knows himself or herself, and is open to the needs of others, sees his or her life as meaningful and valuable, accompanied by joy and peace. Whereas, the low score on the scale indicates that a person’s life lacks a deep relationship with God. A person treats himself and others objectively, and a lack of commitment to others is visible. The life of oneself and others is seen as worthless and meaningless.
- The high score in the subscale of the experience of God’s absence (AG) suggests that the person is striving for greater trust in God, perceiving Him as the one who triggers creative anxiety, and is conducive to discovering the meaning of life. He seeks to get to know himself better and to be more open to others, as well as to gain more knowledge about others. Moreover, the low score in the scale of the experience of God’s absence indicates that man treats God as a preferential value. He does not care about getting to know himself and others better. He is accompanied by a lack of openness to new challenges and a lack of commitment to others.

In addition, the literature points to the multifaceted nature of the Christian religious experience. This scale concerning the experience of God’s presence and God’s absence can be useful to develop a tool for the study of Catholic religious experience taking into account other aspects of it not analyzed so far.

An important advantage of the method is that it concerns the experience of the presence of God and the experience of absence of God within the Catholic religion. The scale is a tool with satisfactory psychometric parameters, taking into account the discriminating power of its item, as well as its reliability and validity.
